# Triple-negative breast cancer and PTEN (phosphatase and tensin homologue)loss are predictors of *BRCA1 *germline mutations in women with early-onset and familial breast cancer, but not in women with isolated late-onset breast cancer

**DOI:** 10.1186/bcr3347

**Published:** 2012-11-02

**Authors:** Sze-Yee Phuah, Lai-Meng Looi, Norhashimah Hassan, Anthony Rhodes, Sarah Dean, Nur Aishah Mohd Taib, Cheng-Har Yip, Soo-Hwang Teo

**Affiliations:** 1 Cancer Research Initiatives Foundation, Sime Darby Medical Centre, 1 Jalan SS12/1A, Subang Jaya, 47500 Selangor, Malaysia; 2 Department of Pathology, Faculty of Medicine, University Malaya Medical Centre, 50603, Kuala Lumpur, Malaysia; 3 Faculty of Applied Sciences, University of the West of England, Bristol, Frenchay Campus, Coldharbour Lane, Bristol BS16 1QY, UK; 4 Breast Cancer Research Unit, University Malaya Cancer Research Institute, Faculty of Medicine, University Malaya Medical Centre, University Malaya, 50603, Kuala Lumpur, Malaysia

## Abstract

**Introduction:**

Given that breast cancers in germline *BRCA1 *carriers are predominantly estrogen-negative and triple-negative, it has been suggested that women diagnosed with triple-negative breast cancer (TNBC) younger than 50 years should be offered *BRCA1 *testing, regardless of family cancer characteristics. However, the predictive value of triple-negative breast cancer, when taken in the context of personal and family cancer characteristics, is unknown. The aim of this study was to determine whether TNBC is a predictor of germline *BRCA1 *mutations, in the context of multiple predictive factors.

**Methods:**

Germline mutations in *BRCA1 *and *BRCA2 *were analyzed by Sanger sequencing and multiple ligation-dependent probe amplification (MLPA) analysis in 431 women from the Malaysian Breast Cancer Genetic Study, including 110 women with TNBC. Logistic regression was used to identify and to estimate the predictive strength of major determinants. Estrogen receptor (ER) and phosphatase and tensin homologue (PTEN) status were assessed and included in a modified Manchester scoring method.

**Results:**

Our study in an Asian series of TNBC patients demonstrated that 27 (24.5%) of 110 patients have germline mutations in *BRCA1 *(23 of 110) and *BRCA2 *(four of 110). We found that among women diagnosed with breast cancer aged 36 to 50 years but with no family history of breast or ovarian cancer, the prevalence of *BRCA1 *and *BRCA2 *mutations was similar in TNBC (8.5%) and non-TNBC patients (6.7%). By contrast, in women diagnosed with breast cancer, younger than 35 years, with no family history of these cancers, and in women with a family history of breast cancer, the prevalence of mutations was higher in TNBC compared with non-TNBC (28.0% and 9.9%; P = 0.045; and 42.1% and 14.2%; P < 0.0001, respectively]. Finally, we found that incorporation of estrogen-receptor and TNBC status improves the sensitivity of the Manchester Scoring method (42.9% to 64.3%), and furthermore, incorporation of *PTEN *status further improves sensitivity (42.9% to 85.7%).

**Conclusions:**

We found that TNBC is an important criterion for highlighting women who may benefit from genetic testing, but that this may be most useful for women with early-onset breast cancer (35 years or younger) or with a family history of cancers. Furthermore, addition of TNBC and PTEN status improves the sensitivity of the Manchester scoring method and may be particularly important in the Asian context, where risk-assessment models underestimate the number of mutation carriers.

## Introduction

Discovery of the breast cancer-predisposition genes *BRCA1 *and *BRCA2 *has enabled us to identify carriers accurately, to target the reduction of risk of breast and ovarian cancers in carriers, and to develop a new generation of targeted therapies (PARP inhibitors) [1]. However, given that deleterious mutations in these genes account for only 1% to 4% of all breast cancer cases across different populations [2] and that genetic testing and genetic counseling have hitherto been relatively expensive, genetic testing for *BRCA1 *and *BRCA2 *has typically been offered only in clinical genetics settings to women who have early-onset breast cancer, and/or to individuals with significant family history of breast and ovarian, or other BRCA-related cancers.

Recently, it was suggested that screening women with early-onset triple-negative breast cancer (TNBC) may be a cost-effective method with which to identify *BRCA1 *mutation carriers in Caucasian women [3-5]. This is because, in the majority of *BRCA1 *carriers, breast tumors have distinctive morphologic features and immunohistochemical phenotypes characteristic of basal-like breast cancers, including negative expression of the estrogen receptor, high expression of basal markers, such as basal cytokeratins CK5/6 and CK14, and loss of tumor-suppressor PTEN [6-8]. Moreover, molecular gene-expression profiling of *BRCA1 *tumors showed that the tumors have significant similarities with the basal-like subtype of breast cancer [9]. Up to 50% of women diagnosed with breast cancer, younger than 50 years, and women who have a family cancer history may have mutations in *BRCA1 *or *BRCA2 *[10]. However, it is notable that although more than 10% women in whom an isolated TNBC develops at younger than 40 years old may have a mutation in *BRCA1 *[3-5], insufficient evidence exists for those aged 41 to 50 years, with no family history of breast or ovarian cancer [11].

The purpose of this study was to determine whether TNBC is an independent criterion for stratifying women with an increased risk of having a *BRCA1 *mutation and to determine whether the addition of immunohistologic features of basal-like breast cancers helps to define a subset of women who are likely to have germline mutations in *BRCA1*.

## Materials and methods

### MyBrCa Breast cancer cohort

The recruitment of breast cancer patients into the Malaysian Breast Cancer Genetic Study (MyBrCa) started in January 2003 at the University Malaya Medical Centre in Kuala Lumpur. All were histopathology-proven breast carcinomas. Histomorphologic and biomarker parameters were retrieved from the histopathology reports. Blood, demographic, and family-history data were collected from breast cancer patients who consented to participate in this study. This study was approved by the ethics committee of University Malaya Medical Centre.

### Analysis of *BRCA1 *and *BRCA2 *

From January 2003 to February 2012, 1,454 breast cancer patients were recruited into the MyBrCa study. Germline DNA samples were screened for *BRCA1 *and *BRCA2 *mutations for all women with (a) early-onset breast cancer (≤35 years of age) (35 with and 96 without family history of breast and ovarian cancer); (b) family history of breast or ovarian cancer in first- and second-degree relatives (193 women); or (c) isolated triple-negative breast cancer diagnosed at between 36 and 50 years old in the absence of family history (47 women). In addition, of the 432 women who were diagnosed aged 36 to 50 years with non-TNBC, 60 women with the highest risk were analyzed (bilateral breast cancer, breast and ovarian cancer in the index patient, family history of breast and ovarian cancer in third-degree or isolated breast cancer (≤45 years of age)). Mutation detection for germline *BRCA1 *and *BRCA2 *mutations was conducted by using direct DNA sequencing and multiple ligation-dependent probe amplification (MLPA), as previously described [12,13].

### Pathologic analysis

For this cohort, patients were classified as having TNBC when we found <10% ER^-^, <10% PR^-^, and 0 or ≤2^+ ^HER2 staining with immunohistochemistry. HER2 was not routinely tested for in all patients prior to 2006, and therefore, in 259 patients, HER2 status was unavailable. Of the 110 women for whom germline *BRCA1 *and *BRCA2 *mutations were analyzed, adequate archived paraffinized invasive tumor tissue for evaluation was available from 32 index patients and two relatives. All cases were tested with immunohistochemistry for cytokeratin 5/6 (D5/16B4; Dako Ltd.), cytokeratin 14 (LL02; Novocastra Labs), and PTEN (6H2.1; Dako). Cytokeratin 5/6 or cytokeratin 14 was defined as positive if >10% of invasive tumor cells showed cytoplasmic staining [14], and PTEN was defined as negative if PTEN staining was undetectable in tumor cells in contrast to adjacent normal stromal cells [6-8]. Description of other pathologic features was conducted as previously described [14].

### Statistical methods

Unconditional multiple linear logistic regression was used to model the probability that the proband was a mutation carrier as a function of her personal and family history and age at diagnosis, as described in [15].

### Manchester score analysis

Manchester score was calculated as previously described [16]. In brief, this system assigns scores depending on the type of cancer and age at diagnosis and developed such that a score of 15 was equivalent to a 10% chance of identifying a *BRCA1 *or *BRCA2 *mutation. The modified method [17-19] includes upward adjustments for TNBC (+4), estrogen-negative (+1), and high-grade invasive cancers (+2), and downward adjustments for human epidermal growth factor receptor 2 (HER2)-positive (-4) or estrogen-receptor positive (-1), lobular (-2), and low-grade noninvasive cancers (-2). For individuals in whom tumor tissue was available for PTEN staining, upward (+1) and downward (-1) adjustments were made for the absence or presence of PTEN staining, respectively.

## Results

### Prevalence of BRCA1 and BRCA2 germline mutations is higher among subsets of women with TNBC compared with non-TNBC

Of the 1,454 breast cancer patients in the MyBrCa Study, 177 (12.2%) had TNBC based on pathology reports. Of these 177 women, 50 older than 50 years developed breast cancer and did not have any family history of breast or ovarian cancer in first- or second-degree relatives and were therefore excluded from the study. Of the remaining 127 women, 63 had already been analyzed because of family history of either breast and/or ovarian cancer (38 individuals, 13 *BRCA1 *and three *BRCA2 *carriers, mutation prevalence, 42.1%) or early-onset breast cancer (25 individuals, seven *BRCA1 *and no *BRCA2 *carriers; mutation prevalence, 28.0%]. Of the remaining 64 women diagnosed at ages 36 through 50 years, but with no family history of breast or ovarian cancer, 47 women were screened for germline mutations in *BRCA1 *and *BRCA2 *genes, and three *BRCA1 *and one *BRCA2 *carriers were identified (mutation prevalence, 8.5%). Overall, of the 110 women who developed TNBC and were analyzed (63 with and 47 without family history of breast and ovarian cancers), 23 *BRCA1 *and 4 *BRCA2 *carriers were identified, giving a mutation prevalence of 24.5% (Table 1).

**Table 1:** Characteristics of women tested for germline *BRCA1 *and *BRCA2 *mutations

Characteristics	Total		*BRCA1*		*BRCA2*		*BRCA1 and -2*	
	
	*n*	(%)	*n*	(%)	*n*	(%)	*n*	(%)
Female breast, age at index diagnosis, years								

≤30	50	11.6	8	16.0	2	4.0	10	20.0
31-40	164	38.1	17	10.4	13	7.9	30	18.3
41-50	144	33.4	8	5.6	8	5.6	16	11.1
>50	73	16.9	4	5.5	5	6.8	9	12.3

Breast or ovarian cancers in family (first and second degree only)								

Female breast	217	50.3	21	9.7	20	9.2	41	18.9
Female ovary	19	4.4	6	31.6	3	15.8	9	47.4

Manchester score								

≤10	205	47.6	5	2.4	6	2.9	11	5.4
11-17	146	33.9	16	11.0	10	6.8	26	17.8
≥18	80	18.6	16	20.0	12	15.0	28	35.0

Ancestry								

Malay	115	26.7	8	7.0	9	7.8	17	14.8
Chinese	248	57.5	15	6.0	16	6.5	31	12.5
Indian	59	13.7	12	20.3	3	5.1	15	25.4
Others	9	2.1	2	22.2	0	0.0	2	22.2

Referral characteristic								

Early onset ≤35 years, regardless of family history	131	30.4	17	13.0	8	6.1	25	19.1
Two cases of breast cancer, one <50 years	126	29.2	10	7.9	11	8.7	21	16.7
Three cases of breast or ovarian cancer	76	17.6	13	17.1	12	15.8	25	32.9
One case of bilateral breast cancer <50 years, in index or first- and second-degree relative	39	9.0	10	25.6	3	7.7	13	33.3
One case of breast and ovarian cancer in same individual in index or first- and second-degree relative	8	1.9	3	37.5	1	12.5	4	50.0
Triple-negative breast cancer, ≤50 years	98	22.7	20	20.4	3	3.1	23	23.5

In total, 431 breast cancer patients were analyzed for germline mutations in *BRCA1 *and *BRCA2 *by DNA sequencing and multiple ligation-dependent probe amplification (MLPA) analysis. Table 1 shows the distribution of women according to their age at diagnosis, family history of breast and ovarian cancer in first- and second-degree relatives, Manchester score and self-declared ethnicity, and the prevalence of *BRCA1 *and *BRCA2 *mutations in each category.

In total, 321 women with non-TNBC were analyzed. This included all women with a family history of breast and/or ovarian cancer (190 individuals, 10 *BRCA1 *and 17 *BRCA2 *carriers, mutation prevalence 14.2%) and women diagnosed at 35 years or younger with no family history of breast and ovarian cancer (71 individuals, three *BRCA1 *and four *BRCA2 *carriers; mutation prevalence, 9.9% (Table 2)). In addition, of the 432 women who were diagnosed aged 36 through 50 years with non-TNBC, 60 women with the highest risk were analyzed (bilateral breast cancer, breast and ovarian cancer in the index patient, family history of breast and ovarian cancer in third degree or isolated breast cancer (aged 45 years or younger)). Of these 60 women, one *BRCA1 *and three *BRCA2 *carriers were found [mutation prevalence, 6.7%]. Overall, of the 321 non-TNBC women analyzed, 14 *BRCA1 *and 24 *BRCA2 *carriers were identified, giving a mutation prevalence of 11.8%.

**Table 2:** Characteristics of women tested for germline *BRCA1 *and *BRCA2 *mutations

Characteristics	Triple negative				Not triple negative				*P *value
	*n *= 110				*n *= 321				
		
	*n*	*BRCA1*	*BRCA2*	Total (%)	*n*	*BRCA1*	*BRCA2*	Total (%)	
With family history									
Early onset, ≤35 years old	6	4	0	4 (66.7)	29	3	4	7 (24.1)	0.063^a^
>35 years old	32	9	3	12 (37.5)	161	7	13	20 (12.4)	0.0005^b^
Overall	38	13	3	16 (42.1)	190	10	17	27 (14.2)	<0.0001^b^
Without family history									
Early onset, ≤35 years old	25	7	0	7 (28.0)	71	3	4	7 (9.9)	0.045^a^
36 to 50 years old	47	3	1	4 (8.5)	60	1	3	4 (6.7)	1.000^a^
Overall	72	10	1	11 (15.3)	131	4	7	11 (8.4)	0.131^b^
All, regardless of family history or age	110	23	4	27 (24.5)	321	14	24	38 (11.8)	0.001^b^
Mean age at diagnosis (years)	40.6				42.0				0.172
Mean number of first-degree relatives	7.2				7.1				0.827
Mean number of affected (breast or ovarian) relatives, first or second degree	0.6				0.8				0.003
Prevalence of *BRCA1 *mutations		20.9%				4.4%			<0.0001^b^
Prevalence of *BRCA2 *mutations			3.6%				7.5%		0.158^b^

^a^Fisher Exact test. ^b^χ^2 ^test. The prevalence of germline mutations in *BRCA1 *and *BRCA2 *(combined) in the 431 breast cancer patients analyzed, in whom 110 developed triple-negative breast cancer, and 321 did not. Family history includes presence of breast or ovarian cancer in first- and second-degree relatives, bilateral breast cancer in the index patient or relative, or breast and ovarian cancer in the same individual in the index patient or relative. *P *values were calculated by using Fisher Exact or χ^2 ^test, and mean values were calculated by using independent *t *test.

We compared the prevalence of *BRCA1 *and *BRCA2 *mutations in the women who developed TNBC and non-TNBC. Of the women with low familial risk of breast and ovarian cancer (diagnosed 36 to 50 with no family history of breast or ovarian cancer], 6.4% and 1.7% of women with TNBC and non-TNBC were found to be *BRCA1 *carriers, respectively, whereas 2.1% and 5.0% were *BRCA2 *carriers. Overall, 8.5% of women with TNBC and 6.7% of women with non-TNBC were found to have germline *BRCA1 *or *BRCA2 *mutations. This suggests that, regardless of the TNBC status, in the absence of family history of breast or ovarian cancer in this age group, a low (<10%) probability exists of having a *BRCA1 *or *BRCA2 *mutation.

By contrast, in two other groups of women, a significantly higher prevalence of *BRCA1 *mutations was found in women who developed TNBC versus non-TNBC. First, of the women in whom breast cancer developed at 35 years or younger, 28.0% of women with TNBC were *BRCA1 *or *BRCA2 *carriers compared with only 9.9% among those who developed non-TNBC (*P *= 0.045). Notably, all 28.0% of women diagnosed with TNBC at younger than 35 years were *BRCA1 *carriers, compared with 4.2% *BRCA1 *and 5.6% *BRCA2 *among women diagnosed with non-TNBC.

Second, of women who had a family history of breast and/or ovarian cancer, 42.1% of women with TNBC were *BRCA1 *or *BRCA2 *carriers compared with 14.2% of those with non-TNBC (*P *= < 0.0001). This is largely because of a difference in prevalence of *BRCA1 *mutations in TNBC compared with non-TNBC (34.2% compared with 5.3%; *P *= < 0.0001), as no significant difference occurred in the prevalence of *BRCA2 *in both subsets of women (7.9% compared with 8.9%; *P *= 1.00). Notably, no significant difference was noted in average age at onset of breast cancer in the index patient, or the mean number of first-degree relatives of women in whom TNBC developed compared with the non-TNBC (seven relatives), but the mean number of affected relatives in the non-TNBC group was higher than that in the TNBC group (0.8 compared with 0.6; *P *= 0.003). This suggests that the higher prevalence of *BRCA1 *mutations in the women in whom TNBC developed compared with women in whom non-TNBC developed is not due to a difference in the age at onset or strength of family history. Taken together, the results suggest that early onset and familial breast cancer patients in whom TNBC develops are more likely to have mutations in the *BRCA1 *genes compared with those in whom non-TNBC develops (24.5% versus 11.8%; *P *= 0.001).

In addition, in this cohort, a marked difference appears in prevalence of *BRCA1 *and *BRCA2 *in the TNBC and non-TNBC patients. TNBC patients are more likely to have *BRCA1 *than *BRCA2 *mutations (20.9% and 4.4%; p < 0.0001), whereas no statistically significant difference is present in *BRCA1 *and *BRCA2 *mutations in non-TNBC patients (3.6% and 7.5%, respectively; *P *= 0.158).

### Logistic regression analyses

Given the possible confounding between the age of diagnosis, subtype of breast cancer, and family history of cancer, it has been demonstrated that the predictive effects of factors should be based on an analysis that takes into account all factors simultaneously [15]. By using binary logistic regression analysis, we found that age at diagnosis of breast cancer, having any family history of breast or ovarian cancer, and having triple-negative breast cancer were associated with a 2.6-fold (confidence interval (CI), 1.4 to 4.8; *P *< 0.003), 3.5-fold (CI, 1.9 to 6.8; *P *< 0.0001), and 3.5-fold (CI, 1.91 to 6.3; *P *< 0.0001) increase in risk of being a *BRCA1 *or *BRCA2 *carrier, respectively. No evidence of interaction effects between these factors was seen. With multiple linear logistic regression analysis, the strongest predictors of mutation status were breast cancer in the proband if age at diagnosis was younger than 35 years (*P *= 0.043), with bilateral breast cancer (*P *= 0.025), with triple-negative breast cancer (*P *< 0.0001), with breast cancer in a first-degree relative if age at diagnosis was younger than 60 years (*P *< 0.01), or ovarian cancer was present in a first- or second-degree relative (*P *< 0.025) (Table 3).

**Table 3:** Logistic regression coefficients (ß), standard errors, and nominal significance levels of potentially predictive factors for *BRCA1 *or *BRCA2 *carriers

Characteristic	β	Standard error	*P*
Baseline	-5.169	1.388	0.000
Proband breast cancer diagnosis ≤35 years	2.791	1.379	0.043
Proband breast cancer diagnosis 36-39 years	2.248	1.365	0.100
Proband breast cancer diagnosis 40-49 years	1.763	1.355	0.193
Proband breast cancer diagnosis 50-59 years	1.065	1.354	0.431
Proband bilateral breast cancer at any age	1.036	0.462	0.025
Proband ovarian cancer at any age	0.238	1.469	0.871
Proband triple-negative breast cancer at any age	1.323	0.331	0.000
For each relative with cancer:			
First degree, breast cancer diagnosis ≤39 years	0.950	0.369	0.010
First degree, breast cancer diagnosis 40-49 years	1.450	0.386	0.000
First degree, breast cancer diagnosis 50-59 years	1.445	0.450	0.001
First degree, breast cancer diagnosis 60+ years	0.207	0.646	0.749
First degree, ovarian cancer at any age	2.556	0.769	0.001
Second degree, breast cancer diagnosis at any age	0.359	0.272	0.187
Second degree, ovarian cancer diagnosis at any age	1.861	0.832	0.025

The multiple linear logistic regression analysis of the probability of breast cancer patients having a germline mutation in the *BRCA1 *or *BRCA2 *gene on the basis of age at diagnosis and family history of breast or ovarian cancers.

The probability that a proband with a given set of personal and family characteristics is a mutation carrier can be estimated from the model fit shown in Table 4. Starting with the log odds score of θ (the baseline coefficient), add the respective regression coefficient (β) for each personal characteristic and the respective regression for each family characteristic multiplied by the number of affected relatives. In this model, an overall score of -1.5 is equivalent to a 15% probability of being a *BRCA1 *or *BRCA2 *carrier. Notably, the overall scores for women with isolated TNBC who were diagnosed aged ≤35 years old, 36 to 39 years old, or 40 to 49 years old, are -1.1, -1.6, and -2.1, respectively, which correlates with a 29%, 14%, and 9% probability of being a *BRCA1 *or *BRCA2 *carrier. The overall scores for women with isolated non-TNBC who were diagnosed at younger than 35 years, 36 to 39 years old, and 40 to 49 years old, are -2.4, -2.9, and -3.4, respectively, which correlates with a 9%, 4%, and 4% probability of being a *BRCA1 *or *BRCA2 *carrier. These results show that women with isolated TNBC have a higher probability of being carriers compared with women with isolated non-TNBC and that, of the women with isolated breast cancers, only women with isolated TNBC diagnosed at younger than 40 years have a greater than 10% probability of having germline mutations in *BRCA1 *or *BRCA2*.

**Table 4:** Overall number of carriers for each predicted range of probability of being a *BRCA1 *or a *BRCA2 *carrier

Overall score	Number of women	Number of carriers	Percentage of carriers
≤3.5	25	0	0
-3.0 to -3.5	49	2	4
-2.5 to -3.0	47	2	4
-2.0 to -2.5	144	13	9
-1.5 to -2.0	58	8	14
-1.0 to -1.5	59	17	29
0 to -1.0	33	11	33
0 to 1	16	12	75

The number of carriers for each predicted range of probability calculated through incorporation of logistic regression coefficients generated in Table 3.

### Pathologic features of *BRCA1 *and non-*BRCA1 *triple-negative breast cancer

We analyzed the pathology reports of *BRCA1 *carriers and compared them with those in *BRCA2 *and non-BRCA carriers. Of the 31 *BRCA1 *index carriers and affected relatives where pathology reports were available, 26 (83.9%) developed TNBC. By contrast, of the 270 *BRCA2 *carriers and non-BRCA carriers, 88 (32.6%) developed TNBC.

Of the 110 TNBC patients included in this study and for whom germline status of *BRCA1 *and *BRCA2 *has been characterized (see earlier), 34 had adequate invasive tumor tissue for evaluation. Slides were cut and immunostained for several markers that have been reported to be useful in defining the basal-like phenotype, including basal cytokeratins CK5/6, CK14, and PTEN. Although some higher grade were present, higher basal cytokeratin, loss of PTEN, high grade of pleomorphism, presence of pushing margins, solid sheets, necrosis, and mitosis in *BRCA1 *carriers compared with non-*BRCA1 *carriers, these differences were not statistically significant (see Additional file 1).

### Inclusion of pathologic features in Manchester scores

To determine whether the addition of pathologic features can define a subset of women who are likely to have germline mutations in *BRCA1 *or *BRCA2*, we calculated the Manchester score for each individual based on the original Manchester score [16] and on the updated Manchester score where pathology was included [18]. Upward adjustments in *BRCA1 *mutation-prediction scores were made for grade 3 ductal cancers, estrogen receptor (ER), and triple-negative tumors, and downward adjustments in the score were made for grade 1 tumors, lobular cancer, ductal or lobular carcinoma *in situ*, noninvasive breast cancer, and ER/HER2 positivity, as described previously [18].

Of the 431 women in this study, 86 were excluded because pathology reports were incomplete. Without adjustment, 72 of 345 women had a Manchester score of ≥15, and this included only 12 of the 28 *BRCA1 *carriers (sensitivity, 42.9%; specificity, 81.1%; PPV, 16.7%). With the adjustment, 82 of the 345 women had a Manchester score of ≥15, and this included 18 of the 28 *BRCA1 *carriers (sensitivity, 64.3%; specificity, 79.8%; PPV, 22.0%) (Table 5). These results show that adjustment in this cohort resulted in 14% increase in the number of tests (72 to 82) and 21% increase in sensitivity (43% to 64%).

**Table 5:** Performance of Manchester Scoring method before and after adjustment with ER status or ER and PTEN status

Manchester Scoring method	Cohort, *n*	Total *BRCA*1 carriers, *n*	No pathology adjustment		With pathology adjustment	
			
			Individuals tested, *n*	*BRCA1 *carriers tested, *n*	Individuals tested, *n*	*BRCA1 *carriers tested, *n*
Adjustment + estrogen-receptor status	345	28	72	12	82	18
Adjustment + estrogen-receptor + PTEN status	26	7	5	3	10	6

The predicted and observed numbers of *BRCA1 *and *BRCA2 *(combined) carriers in women with low (1 to 14) and high Manchester score, either without adjustment for pathologic features, or with adjustment for ER status and other features [17-19], or further adjustment including +1 for loss of PTEN.

In addition, given that *PTEN *loss was associated with *BRCA1 *germline mutations [8], we made a further upward adjustment for *PTEN *loss (+1 point) for all patients for whom PTEN status was available. Without adjustment, five of 26 women had a >10% probability of *BRCA1 *and *BRCA2 *mutations, and this included only three of the seven *BRCA1 *carriers (sensitivity, 42.9%; specificity, 89.5%; PPV, 60.0%). With the adjustment for *PTEN *loss, 10 of the 26 women had a Manchester score ≥15, and this included six of the seven *BRCA1 *carriers (sensitivity, 85.7%; specificity, 78.9%; PPV, 60.0%; Table 5). Although *PTEN *results were available for only a small subset of patients, these results suggest that upward adjustment for *PTEN *may aid the identification of *BRCA1 *carriers.

## Discussion

Our study in an Asian series of triple-negative breast cancer patients demonstrated that up to 24.5% (27 of 110) women have germline mutations in *BRCA1 *(23 of 110) and *BRCA2 *(four of 110), and that the addition of negative estrogen-receptor status and *PTEN *loss improves the sensitivity of the Manchester Scoring method in our Asian cohort.

The results in this study are consistent with that in other cohorts of triple-negative breast cancer patients, in whom 11% to 39% have germline mutations in *BRCA1 *and *BRCA2 *[3,6,20-23], and cohorts of estrogen-receptor-negative breast cancer patients, of whom 24% to 29% have germline mutations in *BRCA1 *and *BRCA2 *[4,6,24,25]. A recent single-institution study showed that 50% of high-risk patients with TNBC had mutations in *BRCA1/2*, but notably, 76% of this cohort had a family history of breast cancer [10]. For all of these series and for our study, *BRCA1 *mutations are more common than *BRCA2 *mutations.

Cost-effectiveness analyses have suggested that mutation testing for all TNBC patients younger than 50 years old may be a cost-effective approach, assuming that 10% to 25% of these patients have *BRCA1 *or *BRCA2 *mutations [5]. However, we find that although TNBC is associated with an increased prevalence of *BRCA1 *and *BRCA2 *mutations among those younger than 35 years old (28.0% in TNBC versus 9.9% in non-TNBC; *P *= 0.045], TNBC is not associated with an increased prevalence of mutations among those aged 36 to 50 years without a family history of breast or ovarian cancer (*BRCA *prevalence of 8.5% and 6.7%, respectively). This may be because *BRCA1 *is a high-penetrance gene and is associated with both early-onset disease and multiple affected family members, and therefore, in the absence of these features, a low prevalence of *BRCA1 *mutations is found, even in TNBC. Further studies with larger population-based datasets are needed to determine the prevalence of *BRCA1 *and *BRCA2 *mutations and the cost-effectiveness of testing women diagnosed with isolated breast cancer, aged 40 to 49 years old.

We find that the prevalence of *BRCA1 *mutations is higher in TNBC compared with non-TNBC in women in whom early-onset breast cancer develops and in women with a family history of breast and ovarian cancer. This effect is largely due to a difference in prevalence of *BRCA1 *mutations and is consistent with the observation that the majority of *BRCA1 *carriers develop early-onset triple-negative basal-like breast tumors that have distinctive morphologic and immunohistochemical characteristics [6-8]. Intriguingly, the significant proportion of TNBC patients who have *BRCA1 *germline mutations in these two subgroups of patients (28.0% and 34.2%) suggests that mutation in *BRCA1 *is a key driver of the development of TNBC. This could be due to the roles of *BRCA1 *in determining cell fate of luminal progenitor cells [26], its effect on transcriptional regulation of ER-gene expression [27], its effect on regulation of mi155 [27], or a combination of these.

Taken together, we suggest that TNBC status may be helpful in stratifying women with a moderate risk of having *BRCA1 *mutations (for example, a weak family history or isolated case of early-onset breast cancer), but may have limited utility in the absence of such features (for example, women with a single case of TNBC aged 40 to 50 years old).

Finally, we find that addition of negative estrogen receptor and TNBC status improves the sensitivity and specificity of the Manchester Scoring method in our cohort and that addition of *PTEN *loss further improves the sensitivity of the method. *PTEN *loss is highly associated with *BRCA1 *breast cancers (28 (82.4%) of 34 of tumor samples from *BRCA1 *carriers showed the loss of PTEN by immunohistochemistry) and can result from gene rearrangements involving DNA double-strand breaks, intragenic inversions on insertions, homozygous deletions, and focalized CNIs [8]. However, given that the data on *PTEN *loss were available on only a small subset of patients, this result requires further validation in a larger cohort of patients. We believe that methods for stratifying the likelihood of carrying a *BRCA1 *and *BRCA2 *mutation that is independent of family history are important, particularly in the Asian context, because the familial and social stigma associated with cancer makes accurate family-history reporting challenging [28].

## Conclusions

In previous studies and in our cohort of TNBC, a significant proportion of women have germline *BRCA1 *mutations. Our study shows that among women with early-onset breast cancer (≤35 years old) and with a family history of breast cancer, a higher prevalence of *BRCA1 *mutations is present in women with TNBC compared with women with non-TNBC. However, no difference in prevalence of *BRCA1 *and *BRCA2 *mutations is noted among women who develop isolated breast cancer aged 36 to 50 (7% to 8% prevalence in both TNBC and non-TNBC). Our study suggests that the current clinical recommendations of offering *BRCA1 *and *BRCA2 *genetic testing, even to women with isolated TNBC younger than 60 years, warrants further analysis.

## Abbreviations

*BRCA1*: breast cancer 1 gene; *BRCA2*: breast cancer 2 gene; CK: cytokeratin; CNI: DNA copy number increase; ER: estrogen receptor; HER2: human epidermal growth factor receptor 2; MyBrCa: Malaysian Breast Cancer Genetic Study; NCCN; National Comprehensive Cancer Network; PR: progesterone receptor; PTEN: phosphatase and tensin homologue; TNBC: triple-negative breast cancer.

## Competing interests

The authors declare that they have no competing interests.

## Authors' contributions

PSY carried out the genetic analyses. LLM, AR, and SD conducted the pathological analyses, and NH conducted the statistical analyses. NAMT and YCH recruited patients and collected clinical data. PSY and TSH conceived the study and drafted the manuscript. All authors read and approved the final manuscript.

## Supplementary Material

Additional file 1 **CK, cytokeratin**. Characteristics of *BRCA1 *carriers Additional File 1 shows the pathologic features of breast cancers in eight *BRCA1 *carriers compared with 26 non-*BRCA1 *carriers. Click here for file
