# Supplementary material for: Triple-negative breast cancer and PTEN (phosphatase and tensin homologue)loss are predictors of BRCA1 germline mutations in women with early-onset and familial breast cancer, but not in women with isolated late-onset breast cancer
Source: Breast Cancer Res. 2012 Nov 2;14(6):R142. doi: 10.1186/bcr3347 (PMC4053128; doi:10.1186/bcr3347)
Supplement: Additional file 1 — CK, cytokeratin. Characteristics of BRCA1 carriers Additional File 1 shows the pathologic features of breast cancers in eight BRCA1 carriers compared with 26 non-BRCA1 carriers. [file bcr3347-S1.DOCX]

**Additional File 1: Characteristics of *BRCA1* carriers**

| **Characteristics** | **BRCA1 carriers (n=8)** | | **Non-BRCA1 carriers (n=26)** | | **P value** |
| --- | --- | --- | --- | --- | --- |
|  | **Number** | **%** | **Number** | **%** |  |
| Mean age at diagnosis | 42 |  | 43.6 |  | 0.773 |
| Size | 3.4 |  | 3.3 |  | 0.914 |
| Grade |  |  |  |  |  |
| 1 | 1 | 12.5 | 3 | 11.5 | 0.372 |
| 2 | 0 | 0.0 | 6 | 23.1 |  |
| 3 | 7 | 87.5 | 17 | 65.4 |  |
| CK5/6 |  |  |  |  |  |
| Positive | 5 | 62.5 | 13 | 65.0 | 1.000 |
| Negative | 3 | 37.5 | 7 | 35.0 |  |
| CK14 |  |  |  |  |  |
| Positive | 6 | 75.0 | 12 | 57.1 | 0.435 |
| Negative | 2 | 25.0 | 9 | 42.8 |  |
| CK5/6 & CK14 |  |  |  |  |  |
| Either positive | 7 | 87.5 | 14 | 70.0 | 0.633 |
| Neither positive | 1 | 12.5 | 6 | 30.0 |  |
| PTEN loss |  |  |  |  |  |
| Present | 6 | 85.7 | 9 | 47.4 | 0.178 |
| Absent | 1 | 14.3 | 10 | 52.6 |  |
| Pleomorphism |  |  |  |  |  |
| Grade 1 | 1 | 12.5 | 2 | 7.7 | 0.308 |
| Grade 2 | 1 | 12.5 | 11 | 42.3 |  |
| Grade 3 | 6 | 75.0 | 13 | 50.0 |  |
| Pushing Margin |  |  |  |  |  |
| Present | 5 | 71.4 | 10 | 38.5 | 0.203 |
| Absent | 2 | 28.6 | 16 | 61.5 |  |
| Solid Sheets |  |  |  |  |  |
| Present | 6 | 75.0 | 13 | 50.0 | 0.257 |
| Absent | 2 | 25.0 | 13 | 50.0 |  |
| Lymphocytic Infiltration |  |  |  |  |  |
| Present | 5 | 62.5 | 18 | 69.2 | 1.000 |
| Absent | 3 | 37.5 | 8 | 30.8 |  |
| Necrosis |  |  |  |  |  |
| Present | 6 | 75.0 | 16 | 61.5 | 0.681 |
| Absent | 2 | 25.0 | 10 | 38.5 |  |
| Mitosis |  |  |  |  |  |
| Grade 1 | 1 | 12.5 | 7 | 26.9 | 0.868 |
| Grade 2 | 2 | 25.0 | 7 | 26.9 |  |
| Grade 3 | 5 | 62.5 | 12 | 46.2 |  |
| Tubules |  |  |  |  |  |
| Grade 1 | 0 | 0.0 | 0 | 0.0 | 1.000 |
| Grade 2 | 0 | 0.0 | 1 | 3.8 |  |
| Grade 3 | 8 | 100.0 | 25 | 96.2 |  |

Additional File 1 shows the pathological features of breast cancers in 8 BRCA1 carriers compared to 26 non-BRCA1 carriers.
